# Developing Librarian Competencies for the Digital Age

**DOI:** 10.5195/jmla.2018.506

**Published:** 2018-10-01

**Authors:** Elizabeth Connor

**Affiliations:** Daniel Library, The Citadel, Charleston, SC

Despite the generic book title, this book is part of the series of staff development books published by the Medical Library Association with Rowman & Littlefield. The changes and challenges faced by health sciences librarians require the skills described in this practical and highly readable work. Chapter contributors represent organizations in the District of Columbia, Illinois, Indiana, Kansas, Maryland, Michigan, North Carolina, Ohio, South Carolina, Utah, and Virginia.

For librarians who are new to the field, chapter 1 provides a concise introduction to historical foundations and provides a glimpse into the skills required of present-day librarians: adaptability, flexibility, multitasking, and creativity. This first chapter sets the stage for the subsequent chapters that delve into modern collections (chapter 2); how information is organized (chapter 3); technical knowledge related to communicating, teaching, marketing, and computing (chapter 4); reference services and tools (chapter 5); research skills (chapter 6); distance learners (chapter 7); political and strategic aspects of library administration (chapter 8); core competencies (chapter 9); and glimpses into how future library users might view the library (chapter 10).

The work could have been improved with detailed biographical notes for each of the contributors; for example, in some cases, it was not clear where the author’s organization was situated. The four-page index could have been more detailed, as this work is likely to be consulted for specific topics rather than read straight through. For example, the concept of research life cycle is illustrated in chapter 2 and described in depth in chapter 6, but the term “research life cycle” is not in the index.

The book is highly recommended for all levels of health sciences librarians. Librarians at the entry level and at mid-career and later will find much here to appreciate, adopt, and apply.

